# Association Between Mediterranean Diet Adherence and Intuitive and Mindful Eating in Turkish Young Adults

**DOI:** 10.3390/nu18020196

**Published:** 2026-01-07

**Authors:** Hande Ongun Yilmaz, Sedat Arslan, Kevser Tari Selcuk, Salim Yilmaz

**Affiliations:** 1 Department of Nutrition and Dietetics, Bandirma Onyedi Eylul University, 10250 Balikesir, Türkiye; hyilmaz@bandirma.edu.tr (H.O.Y.); kselcuk@bandirma.edu.tr (K.T.S.); 2 Department of Nutrition and Dietetics, Bursa Uludag University, 16059 Bursa, Türkiye; sedatarslan@uludag.edu.tr; 3 Department of Healthcare Management, Faculty of Health Sciences, Acibadem Mehmet Ali Aydinlar University, 34752 Istanbul, Türkiye; 4 Department of Healthcare Management, Graduate School of Health Sciences, Acibadem Mehmet Ali Aydinlar University, 34752 Istanbul, Türkiye

**Keywords:** Mediterranean diet, intuitive eating, mindful eating, eating behaviors, hierarchical regression, Turkish young adults

## Abstract

**Background/Objectives**: This study aimed to examine the association between Mediterranean diet adherence and adaptive eating behaviors, specifically intuitive eating and mindful eating, among Turkish young adults. **Methods**: This cross-sectional study included 2293 young adults aged 18–34 years who completed an online survey between December 2023 and March 2024. Data were collected using the Mediterranean Diet Adherence Scale (MEDAS), Intuitive Eating Scale-2 (IES-2), and Mindful Eating Questionnaire (MEQ-30). One-way ANOVA compared eating behavior scores across adherence groups. Hierarchical multiple regression analyses examined the unique contribution of MEDAS scores after controlling for demographic, socioeconomic, health, lifestyle, and nutritional factors. **Results**: Among the participants, 64.5% demonstrated low, 27.0% moderate, and 8.4% high Mediterranean diet adherence. ANOVA revealed significant differences in both IES-2 and MEQ-30 scores across groups. In hierarchical regression, MEDAS significantly predicted intuitive eating (B = 0.023, *p* = 0.004, contributing 10.72% to explained variance) and mindful eating (B = 0.776, *p* = 0.001, contributing 13.61%) after controlling for all covariates. BMI emerged as the strongest predictor for both outcomes, with divergent associations: negative for intuitive eating and positive for mindful eating. Final models explained 5.8% and 6.2% of variance in IES-2 and MEQ-30, respectively. **Conclusions**: Mediterranean diet adherence demonstrated significant positive associations with both intuitive and mindful eating behaviors, independent of multiple confounders. Although effect sizes were modest, these findings suggest that promoting Mediterranean dietary patterns may complement interventions aimed at fostering adaptive eating behaviors. The divergent BMI associations warrant further investigation.

## 1. Introduction

The Mediterranean diet, which is one of the most emphasized healthy eating models, and one of its main features is nutritional diversity, constitutes an example of an adequate and balanced diet. The Mediterranean diet is a nutritional model characterized by a diet rich in plant foods such as fruits and vegetables, whole grains, legumes, oilseeds, and olive oil. It also includes moderate to high consumption of fish and seafood, moderate consumption of dairy products, eggs, poultry, and wine, and low consumption of red meat and processed foods [1]. There is a large body of scientific evidence demonstrating the importance of the Mediterranean diet in the prevention of chronic diseases and health maintenance, as it provides significant health and nutritional benefits [2,3,4,5,6]. Studies have demonstrated that higher adherence to the Mediterranean diet is linked with weight management, including decreased risk of overweight and obesity, reduced waist circumference, and a lower likelihood of abdominal obesity. Moreover, adherence to this diet has been shown to be effective in preventing chronic diseases [7,8,9,10,11,12,13].

Eating behavior, characterized by a complex interaction of individual, socio-cultural, psychological, environmental, and economic factors, can both positively and negatively impact health. Interventions to change individuals’ eating behaviors focus on promoting healthy eating patterns that help prevent and manage chronic diseases [14]. In recent years, there has been increasing attention on the concepts of intuitive eating and mindful eating, which are mindfulness-based approaches argued to be effective in maintaining healthy nutrition and body weight beyond traditional dietary patterns [15,16,17].

Intuitive eating, which is expressed as eating in response to and relying on physiological hunger and satiety cues, is defined as individuals’ knowledge of the amount and type of food the body needs, developed in response to body weight control without a specific health problem [15,18]. Intuitive eating, which refers to an approach to eating based on instinct, involves listening to the body’s needs by paying attention to feelings of hunger and satiety, and eating based on these feelings. Intuitive eating contributes to maintaining regular nutrition, cultivating a positive body image, and enhancing emotional well-being by facilitating healthy communication with one’s body and guiding eating habits accordingly [19,20]. The basic approach of intuitive eating is that the individual instinctively makes choices to ensure nutritional balance without restricting food diversity. In intuitive eating, after adequate food intake is achieved, the meal can be stopped without over-saturation. It is reported that intuitive eating may be an appropriate approach for the prevention and treatment of obesity [15,21].

Mindful eating is defined as eating by realizing how and why eating behavior occurs rather than what is eaten, internalizing the concept of physical hunger–fullness, and being aware of the effect of emotions and thoughts, focusing on the food to be consumed at that moment without being affected by environmental factors and without judging food choices. Mindful eating can slow down the rate of eating, reduce food cravings, and help to achieve weight control [16,22]. The literature has examined how interventions aimed at promoting intuitive eating and mindful eating impact body weight, dietary content, and quality. It has been reported that these mindfulness-based approaches are associated with positive effects on weight control, obesity, and an improvement in diet quality [16,17,23,24,25,26].

The Mediterranean diet, which is known to have positive effects on health, is a nutritional model that emphasizes nutritional diversity and does not prioritize restriction. The Mediterranean diet is also a lifestyle that recommends low levels of stress through physical activity and social interaction. Therefore, individuals with high adherence to the Mediterranean diet are also expected to have high levels of intuitive eating and mindful eating. Although there are studies examining the relationship between Mediterranean diet adherence and mindful eating in the literature [27,28], there are a very limited number of studies examining the relationship between Mediterranean diet adherence and both intuitive eating and mindful eating [29]. Young adulthood represents a critical period for establishing long-term dietary habits and eating behavior patterns, yet this population remains understudied in this context. Hence, this study aimed to evaluate the relationship between adherence to the Mediterranean diet and intuitive eating and mindful eating in young adults.

## 2. Materials and Methods

### 2.1. Study Design and Sample

The research is cross-sectional. The population of the study consisted of young adults aged 18–34 living in Türkiye. This age range corresponds to the “young adult” life stage as defined by the US Centers for Disease Control and Prevention (CDC) and commonly used in epidemiological research [30]. The minimum sample size required for the research was calculated as 1821 individuals with 99% power, α = 0.05 significance level, and d = 0.10 effect size using the G*Power 3.1.9.7 program [31]. Considering the possibility of missing data within the scope of the research, it was aimed to reach 2367 people by taking 30% more. The research group was determined using convenience sampling through social media and group communication applications between December 2023 and March 2024.

The inclusion criteria were (a) young adults aged 18–34 years, (b) currently residing in Turkey, (c) voluntary participation with informed consent, and (d) completion of the online survey. The exclusion criteria were (a) self-reported diagnosis of psychiatric illness, as psychiatric disorders—particularly depression, anxiety, and eating disorders—are strongly associated with both dietary patterns and eating behaviors, which could confound the relationships under investigation [32,33,34]; (b) following a special diet under dietitian supervision, which could independently influence both Mediterranean diet adherence and eating behaviors; (c) presence of food allergies or intolerances that may restrict dietary choices; and (d) pregnancy or breastfeeding, which alter nutritional requirements and eating patterns.

Among the 2748 individuals who initially participated, 2293 met the eligibility criteria after excluding those diagnosed with psychiatric illness (*n* = 6), following a special diet with a dietitian (*n* = 18), having food allergies or intolerances (*n* = 2), being pregnant or breastfeeding (*n* = 2), and aged over 34 years (*n* = 47).

### 2.2. Data Collection

Prior to data collection, individuals were informed about the purpose and scope of the study, and those included in the study were asked to approve the informed consent form at the beginning of the online survey form. The data of the study were collected online with a questionnaire form consisting of four sections. The first part of the questionnaire included the Descriptive Information Form, the second part included the Mediterranean Diet Adherence Scale (MEDAS), the third part included the Intuitive Eating Scale-2 (IES-2), and the fourth part included the Mindful Eating Questionnaire (MEQ-30).

Descriptive Information Form: It was prepared by the researchers and consisted of questions about the participants’ socio-demographic characteristics (age, gender, educational status, employment status, and perceived income level), health status (chronic disease diagnosis), lifestyle behaviors, dietary habits (number of daily meals, breakfast consumption, and daily water intake), and anthropometric measurements (height, body weight). Height and body weight were self-reported and used to calculate body mass index (BMI, kg/m^2^).

Lifestyle behaviors were assessed as follows: Smoking status was categorized as “yes” for participants who reported smoking at least one cigarette per day, and “no” otherwise. Alcohol consumption was assessed with a single item asking whether participants consume alcohol; those who reported any alcohol consumption, regardless of frequency or quantity, were categorized as “yes.” This binary classification does not distinguish between occasional (e.g., a few times per year) and regular drinkers. Physical activity was categorized as “yes” for participants who reported engaging in regular physical activity, and “no” otherwise.

Mediterranean Diet Adherence Scale (MEDAS): The 14-item Mediterranean Diet Adherence Scale, which determines the adherence of individuals to the Mediterranean diet, was developed by Martínez-González et al. [12] and validated by Schröder et al. [35]. The Turkish validity and reliability of the scale were assessed by Pehlivanoğlu et al. [36]. In the scale consisting of a total of 14 items, a score of 1 or 0 is obtained for each question asked according to the amount of consumption, and the total score is calculated between 0 and 14. A total score of 7 or higher indicates an acceptable degree of compliance with the Mediterranean diet, while a score of 9 or higher indicates strict compliance. The Cronbach α coefficient of the scale was reported as 0.829 [36].

Intuitive Eating Scale-2 (IES-2): The Intuitive Eating Scale-2 was developed by Tylka and Kroon Van Diest [37] as a revised version of the original Intuitive Eating Scale [38], and its Turkish validity and reliability study was conducted by Baş et al. [39]. There are a total of 23 questions on the scale, which examines intuitive eating in 4 sub-dimensions: unconditional permission to eat (eating when hungry and craving certain foods), eating due to physical rather than emotional reasons, eating due to hunger and satiety signals (determining when and how much to eat), and body-nutrient choice compatibility. In the evaluation of the scale with 5-point Likert-type questions, responses are rated as follows: “strongly disagree” 1, “disagree” 2, “undecided” 3, “agree” 4, and “strongly agree” 5. Scoring was performed according to the original validation study, with relevant items reverse-scored as specified [39]. In scoring, a numerical value is obtained by dividing the total score of the scale and each sub-dimension by the number of questions covered by the total score. A higher score on both the total scale and its sub-dimensions indicates greater intuitive eating. The Cronbach α coefficient of the scale was reported as 0.82 [39].

Mindful Eating Questionnaire (MEQ-30): The Mindful Eating Questionnaire was originally developed by Framson et al. [40] to measure awareness and attention given to the eating experience, and the Turkish validity and reliability study was conducted by Köse et al. [22]. The scale consists of 30 items in total and 7 sub-dimensions, including disinhibition, emotional eating, eating control, focusing, eating discipline, awareness, and interference. It is rated on a 5-point Likert scale. In the evaluation of the scale, responses to the questions are rated as follows: “never” 1, “rarely” 2, “sometimes” 3, “often” 4, and “always” 5 points. Scoring was performed according to the original validation study, with relevant items reverse-scored as specified [22]. A higher total scale score or scores on the sub-dimensions indicate greater mindful eating. The Cronbach α coefficient of the scale was reported as 0.733 [22].

### 2.3. Hierarchical Regression Modeling Approach and Model Assumptions Testing

Hierarchical multiple regression analysis was employed to examine the unique contribution of Mediterranean diet adherence (MEDAS score) to intuitive eating (IES-2) and mindful eating (MEQ-30) after controlling for potential confounders. The hierarchical approach allows for a systematic examination of how different categories of variables contribute to the explained variance. By entering MEDAS in the final block, we can determine its incremental predictive validity beyond established correlates of eating behaviors. Variables were entered in six theoretically ordered blocks:

- Block 1 (Demographic): age, gender—fundamental demographic characteristics;
- Block 2 (Socioeconomic): education, employment status, income level, marital status;
- Block 3 (Health): chronic disease status, BMI-health-related factors;
- Block 4 (Lifestyle): smoking, alcohol consumption, physical activity;
- Block 5 (Nutrition habits): number of daily meals, breakfast consumption, daily water intake;
- Block 6 (Main predictor): MEDAS score—Mediterranean diet adherence.

This structure places MEDAS in the final block to assess its unique contribution after accounting for all other relevant factors. The approach follows the principle of entering known predictors before the variable of primary interest. Prior to conducting hierarchical regression analyses, all assumptions of multiple linear regression were systematically evaluated for both outcome variables (Table 1).

Residual normality was assessed using skewness, kurtosis, and the Anderson–Darling test. For the IES-2 model, skewness (0.374) and excess kurtosis (−0.379) were within acceptable limits (|value| < 1). For the MEQ-30 model, skewness was excellent (−0.006), while excess kurtosis (1.34) was marginally elevated but within a tolerable range for large samples. The Anderson–Darling test formally rejected normality for both models (IES-2: A = 7.61, *p* < 0.001; MEQ-30: A = 5.80, *p* < 0.001); however, this is expected in large samples (*n* = 1750) where the test is overly sensitive to minor deviations. Given acceptable skewness and kurtosis values, and the robustness of regression to mild non-normality with large samples, where the central limit theorem ensures robust parameter estimation, the normality assumption was considered adequately met. The Breusch–Pagan test revealed significant heteroscedasticity in both models (IES-2: BP = 49.64, *p* < 0.001; MEQ-30: BP = 30.21, *p* = 0.011), necessitating the use of heteroscedasticity-consistent (HC3) robust standard errors for all analyses. Variance Inflation Factors (VIFs) were examined for all predictors. The maximum VIF value was 1.77, well below the conventional threshold of 5, indicating no problematic multicollinearity among predictors. The Durbin–Watson statistic was 1.80 for IES-2 and 1.90 for MEQ-30, both within the acceptable range (1.5–2.5), suggesting no problematic autocorrelation in the residuals. The Rainbow test for linearity was non-significant for IES-2 (F = 1.01, *p* = 0.418) but significant for MEQ-30 (F = 1.13, *p* = 0.032), suggesting potential non-linearity. To address this, quadratic terms for MEDAS were tested in both models. Neither quadratic term was significant (IES-2: F = 0.04, *p* = 0.846; MEQ-30: F = 2.46, *p* = 0.117), and AIC comparisons favored the linear models (IES-2: AIC_linear_ = 3143.95 vs. AIC_quadratic_ = 3145.91; MEQ-30: AIC_linear_ = 14,930.52 vs. AIC_quadratic_ = 14,930.04). Therefore, linear specifications were retained for both outcomes.

### 2.4. Data Analysis

Statistical analyses were performed using R version 4.4.2 with the following packages: car, lmtest, sandwich, moments, nortest, ggplot2, and relaimpo. Descriptive statistics (frequencies, percentages, means, and standard deviations) were calculated for all variables. The normality of continuous variables was assessed using skewness and kurtosis coefficients, with values within the range of ±1 considered indicative of an acceptable normal distribution. One-way ANOVA was employed to compare mean scores of the Intuitive Eating Scale-2 (IES-2) and Mindful Eating Questionnaire (MEQ-30) across Mediterranean diet adherence groups (low: MEDAS ≤ 6; moderate: MEDAS 7–8; high: MEDAS ≥ 9), with Tukey HSD post hoc tests used to identify specific group differences. Hierarchical multiple regression analysis was conducted to examine the unique contribution of Mediterranean diet adherence to eating behaviors after controlling for potential confounders. Variables were entered in six theoretically ordered blocks. Prior to regression analyses, assumptions were systematically evaluated, including normality (skewness, kurtosis, Anderson–Darling test), homoscedasticity (Breusch–Pagan test), multicollinearity (VIF < 5), independence of residuals (Durbin–Watson statistic within 1.5–2.5), and linearity (Rainbow test). Quadratic terms were tested to assess potential non-linear relationships. Due to significant heteroscedasticity in both models, heteroscedasticity-consistent robust standard errors (HC3) were employed for all analyses. Model comparisons were evaluated using R^2^, adjusted R^2^, ΔR^2^, F-change statistics, AIC, and BIC. The relative importance of predictors was assessed using the Lindeman–Merenda–Gold (LMG) method to decompose R^2^ into individual predictor contributions. Wald tests were conducted to confirm the significance of MEDAS contribution in each model. The significance level was set at *p* < 0.05 for all statistical tests.

### 2.5. Ethical Approval

The study was conducted in accordance with the ethical principles of the Declaration of Helsinki, and approval was obtained from Bandirma Onyedi Eylul University Health Sciences Non-Interventional Research Ethics Committee (2023-195).

## 3. Results

Table 2 shows the descriptive characteristics of the study sample. The mean age of the participants was 23.51 ± 3.27 years (range: 18–34), 59.0% were female, 83.3% were single, and 82.6% had a university degree or higher. Regarding employment status, 49.4% were students, 35.8% were employed, and 14.8% were unemployed. More than half of the participants (52.0%) reported that their income was equal to their expenditure. Among the participants, 10.2% reported having been diagnosed with one or more chronic diseases. Regarding lifestyle behaviors, 35.2% were current smokers, 35.1% reported any alcohol consumption, and 31.3% engaged in regular physical activity. Concerning dietary habits, 43.1% of the participants consumed three main meals daily, 58.7% had breakfast regularly, and 27.4% drank 10 or more glasses of water daily. The mean BMI was 22.93 ± 3.79 kg/m^2^, with 64.4% classified as normal weight (Table 2).

The mean MEDAS score was 5.83 ± 1.87. According to the cut-off point of the scale, 27.0% of the study group achieved acceptable adherence to the Mediterranean diet, and 8.4% achieved strict adherence. The mean scores of the IES-2 total and unconditional permission to eat, eating due to physical rather than emotional reasons, eating due to hunger and satiety signals, and body–food choice compatibility subscales were 3.25 ± 0.59, 3.23 ± 0.61, 3.29 ± 0.75, 3.24 ± 1.09, and 3.18 ± 1.11, respectively. The mean scores of the MEQ-30 total and disinhibition, emotional eating, eating control, focusing, eating discipline, awareness, and interference subscales were 82.64 ± 17.49, 14.25 ± 3.27, 13.98 ± 3.58, 10.95 ± 3.11, 13.27 ± 3.51, 10.89 ± 2.74, 14.13 ± 3.56, and 5.22 ± 1.98, respectively (Table 3).

Table 4 shows the mean scores of IES-2 and MEQ-30 according to adherence to the Mediterranean diet. For overall intuitive eating (IES-2), participants with high adherence scored significantly higher than those with low adherence (F = 6.59, *p* < 0.001). However, the subscale analysis revealed a more nuanced pattern. Unconditional permission to eat showed an inverse relationship, with low-adherence participants scoring higher than moderate and high-adherence groups, and moderate adherence higher than high adherence (F = 28.13, *p* < 0.001). In contrast, eating based on hunger and satiety signals (F = 17.26, *p* < 0.001) and body–food choice compatibility (F = 30.46, *p* < 0.001) demonstrated positive associations, with significant differences across all pairwise comparisons. Eating for physical rather than emotional reasons did not differ significantly across groups (F = 2.17, *p* = 0.115). For mindful eating (MEQ-30), overall scores increased significantly with higher Mediterranean diet adherence (F = 9.73, *p* < 0.001), with both moderate and high-adherence groups scoring higher than the low-adherence group. This pattern was primarily driven by the disinhibition (F = 25.53, *p* < 0.001) and emotional eating (F = 41.65, *p* < 0.001) subscales, which showed significant differences across all pairwise comparisons. Eating discipline also showed a significant effect (F = 5.14, *p* = 0.006), with only the high-adherence group differing from the low-adherence group. Focusing also reached significance (F = 3.01, *p* = 0.049), with the moderate-adherence group scoring higher than the low-adherence group. Eating control (F = 0.33, *p* = 0.717), awareness (F = 2.98, *p* = 0.051), and interference (F = 2.39, *p* = 0.092) subscales did not show significant group differences. These findings suggest that Mediterranean diet adherence is differentially associated with specific components of intuitive and mindful eating, rather than uniformly affecting all dimensions of these constructs.

Hierarchical multiple regression analysis was conducted to examine the unique contribution of Mediterranean diet adherence to intuitive eating after controlling for demographic, socioeconomic, health, lifestyle, and nutritional factors. Heteroscedasticity-consistent robust standard errors (HC3) were employed due to significant Breusch–Pagan test results. All six blocks contributed significantly to the model, with the final model explaining 5.8% of the variance in IES-2 scores (R^2^ = 0.058, Adjusted R^2^ = 0.049, F_(16, 1733)_ = 6.61, *p* < 0.001). The health block (Block 3) contributed the largest increment in explained variance (ΔR^2^ = 0.022, F = 19.66, *p* < 0.001), followed by nutrition habits (Block 5: ΔR^2^ = 0.010, F = 6.20, *p* < 0.001) and socioeconomic factors (Block 2: ΔR^2^ = 0.010, F = 3.48, *p* = 0.004). BMI emerged as the strongest individual predictor, accounting for 40.85% of the total explained variance (B = −0.025, 95% CI [−0.033, −0.016], *p* < 0.001), indicating that each one-unit increase in BMI was associated with a 0.025-point decrease in intuitive eating scores. Breakfast consumption was the second most important predictor (14.31% of R^2^; B = 0.109, 95% CI [0.049, 0.169], *p* < 0.001), with breakfast consumers scoring significantly higher on intuitive eating. The MEDAS score significantly predicted intuitive eating after controlling for all covariates (B = 0.023, 95% CI [0.008, 0.039], *p* = 0.004), contributing 10.72% to the explained variance. This indicates that each one-point increase in Mediterranean diet adherence was associated with a 0.023-point increase in IES-2 scores, and the Wald test confirmed the robustness of this contribution (F_(1, 1733)_ = 8.53, *p* = 0.004). Additional significant predictors included education level (B = −0.087, *p* = 0.032), with non-university educated participants showing lower intuitive eating; smoking (B = −0.067, *p* = 0.038), with smokers demonstrating lower scores; alcohol use (B = 0.068, *p* = 0.035), with alcohol consumers demonstrating higher scores; and number of daily meals (B = −0.034, *p* = 0.012), indicating a negative association between meal frequency and intuitive eating. Employment status (student vs. employed, unemployed vs. employed) did not significantly predict intuitive eating (Table 5).

The other hierarchical multiple regression model analysis was performed to evaluate the predictors of mindful eating, with heteroscedasticity-consistent robust standard errors (HC3) employed due to significant Breusch–Pagan test results (BP = 30.21, *p* = 0.011). The final model explained 6.2% of the variance in MEQ-30 scores (R^2^ = 0.062, Adjusted R^2^ = 0.054, F_(16, 1733)_ = 7.21, *p* < 0.001). The health block (Block 3) demonstrated the largest contribution to explained variance (ΔR^2^ = 0.028, F = 25.05, *p* < 0.001), substantially exceeding all other blocks, followed by socioeconomic factors (Block 2: ΔR^2^ = 0.011, F = 4.04, *p* = 0.001) and nutrition habits (Block 5: ΔR^2^ = 0.006, F = 3.90, *p* = 0.009). BMI was again the strongest predictor, accounting for 29.66% of the explained variance (B = 0.768, 95% CI [0.521, 1.015], *p* < 0.001). Notably, and in contrast to intuitive eating, higher BMI was associated with higher mindful eating scores, with each one-unit BMI increase corresponding to a 0.768-point increase in MEQ-30 scores. Gender emerged as the second strongest predictor (14.15% of R^2^; B = −4.376, 95% CI [−6.345, −2.407], *p* < 0.001), with males scoring approximately 4.4 points lower than females on mindful eating. The MEDAS score was the third most important predictor, contributing 13.61% to the explained variance (B = 0.776, 95% CI [0.304, 1.247], *p* = 0.001). Each one-point increase in Mediterranean diet adherence was associated with a 0.78-point increase in MEQ-30 total score, and the Wald test confirmed this significant contribution (F_(1, 1733)_ = 10.38, *p* = 0.001). A notable finding was that student status significantly predicted higher mindful eating scores compared to employed individuals (B = 3.200, 95% CI [0.872, 5.527], *p* = 0.007), contributing 10.73% to the explained variance. Other significant predictors included chronic disease status (B = 3.315, *p* = 0.014), with those having chronic conditions scoring higher; alcohol use (B = 2.475, *p* = 0.006); and number of daily meals (B = 0.900, *p* = 0.024). Unlike the previous analysis with the full sample, age (B = −0.071, *p* = 0.721) and education (B = −1.717, *p* = 0.170) were not significant predictors in this young adult sample, suggesting that these associations may be driven by age heterogeneity. Similar to the IES-2 model, breakfast consumption was not a significant predictor of mindful eating (B = 0.446, *p* = 0.616), highlighting differential predictive patterns across these eating behavior constructs (Table 6).

## 4. Discussion

This study aimed to investigate the relationship between adherence to the Mediterranean diet and eating behaviors in young adults. Our findings revealed that both intuitive eating and mindful eating increased as adherence to the Mediterranean diet increased, emphasizing the positive impact of healthy dietary patterns on individuals’ eating behaviors.

Numerous studies have reported that adherence to the Mediterranean diet is effective in preventing chronic diseases such as obesity, diabetes, cardiovascular diseases, and certain types of cancer [2,3,4,5,6,7,8,10,41,42]. In our study, 64.5% of young adults demonstrated low adherence to the Mediterranean diet, while only 8.4% achieved high adherence. A systematic review by Damigou et al. [43] reported moderate Mediterranean diet adherence worldwide, with higher rates observed in European countries, particularly Mediterranean regions. Recent studies among Turkish adults have yielded diverse results: Çatak et al. [44] reported approximately 58% low adherence, Yassıbaş et al. [45] found 25%, and Bakırhan et al. [46] reported 19%. These differences may be attributable to variations in measurement tools, sample characteristics (our study focused specifically on young adults aged 18–34 years with predominantly university education), and regional dietary patterns across Türkiye.

Mindfulness-based approaches have gained popularity as interventions for disordered eating and weight management. Intuitive eating aims to improve individual health and promote sustainable changes in one’s relationship with food [25] by enabling individuals to trust their hunger and satiety cues as an alternative to restrictive dietary models [24]. Ayyıldız Atak [29] reported a positive association between Mediterranean diet adherence and intuitive eating behaviors in Türkiye. Consistent with this, our study found that Mediterranean diet adherence positively predicted intuitive eating, supporting research suggesting that individuals with high intuitive eating tendencies consume more nutritious foods and exhibit healthier eating habits [23,24,25]. This relationship may be explained by the Mediterranean diet being a lifestyle that encourages healthy food selection and healthy aging [36].

The IES-2 subscale analysis revealed nuanced patterns. The “eating based on hunger and satiety signals” subscale measures how accurately individuals recognize and respond to hunger and satiety cues, including whether they eat when hungry and stop when full. The “body–food choice compatibility” subscale assesses whether individuals accurately identify their body’s nutritional needs and make food choices accordingly [47]. Individuals with low Mediterranean diet adherence scored significantly lower on both subscales compared to those with moderate and high adherence (*p* < 0.001), suggesting that Mediterranean diet adherence may protect against eating beyond physiological requirements. Conversely, the “unconditional permission to eat” subscale showed an inverse pattern: low-adherence participants scored significantly higher than moderate and high-adherence groups. This suggests that the Mediterranean diet model, which emphasizes healthy food choices rather than energy restriction, may reduce hedonic eating tendencies.

Mindful eating represents another noteworthy approach for addressing irregular eating patterns. It involves paying attention to the eating experience, focusing on how and why eating behavior occurs rather than merely what is consumed [28,48]. The MEQ-30 assesses eating behaviors across seven subscales: disinhibition (self-restraint and control over food quantity and timing), emotional eating (how emotions affect eating), eating control (pace and process regulation), focusing (attention to food and taste), eating discipline (conscious planning and preparation), awareness (hunger–fullness recognition and nutritional knowledge), and interference (coping with sensory distractions) [22]. Consistent with Ayyıldız Atak [29], who found positive associations between Mediterranean diet adherence and all MEQ-30 dimensions, our study revealed that low-adherence participants had significantly lower scores on disinhibition, emotional eating, and eating discipline subscales. This may reflect that individuals with low Mediterranean diet adherence tend to prefer processed foods high in fat, sugar, and salt, which can increase disinhibition and provoke emotional eating. Additionally, the Mediterranean diet typically involves unprocessed foods requiring more preparation time, potentially fostering meal planning skills and eating discipline.

A noteworthy finding was the divergent association of BMI with the two eating behavior constructs: BMI negatively predicted intuitive eating (B = −0.025, *p* < 0.001) but positively predicted mindful eating (B = 0.768, *p* < 0.001). This seemingly paradoxical pattern becomes interpretable when considering conceptual distinctions between these constructs. Intuitive eating emphasizes eating in response to internal physiological cues, whereas mindful eating includes dimensions of deliberate cognitive control. Keirns and Hawkins [49] demonstrated that the intuitive eating-body image relationship progressively weakens with increasing BMI, attributing this to dysregulation of hunger and satiety signals associated with excess adiposity. Additionally, individuals with higher BMI are more likely to experience internalized weight stigma, which predicts external food rules and dietary restraint [50]; repeated dieting attempts may progressively disconnect individuals from internal physiological cues. Conversely, the positive BMI-mindful eating association may reflect compensatory cognitive strategies: individuals disconnected from intuitive body signals may rely more heavily on deliberate, cognitively controlled eating behaviors as weight management strategies, consistent with restraint theory [51]. Our subscale findings partially support this interpretation, as the MEQ-30 “eating control” subscale showed no association with Mediterranean diet adherence (F = 0.33, *p* = 0.717), suggesting cognitive eating control operates independently of dietary pattern quality. These findings imply that interventions for individuals with higher BMI might benefit from approaches that restore connection to internal hunger and satiety cues rather than solely reinforcing cognitive control strategies.

Intuitive eating and mindful eating are complex behavioral constructs shaped by numerous factors, including personality traits, emotional regulation capacity, body image, past dieting history, cultural norms around food, family eating environment during childhood, and socioeconomic food access—most of which were beyond the scope of our assessment. Given this multifactorial nature, expecting any single dietary pattern to account for large portions of behavioral variance would be unrealistic. Studies examining predictors of intuitive eating have reported similar or lower predictive values; Bruce and Ricciardelli [20] found that psychological and demographic variables explained 4–12% of intuitive eating variance across studies [20,52]. Our findings that Mediterranean diet adherence ranked as the third most important predictor in both models—after BMI and breakfast consumption for IES-2, and after BMI and gender for MEQ-30—suggest a meaningful relationship that persists after accounting for numerous confounders. The consistency of this association between two distinct eating behavior constructs strengthens confidence in its robustness. From a public health perspective, even modest individual-level effects can translate into meaningful population-level shifts when dietary interventions reach large numbers of people.

Our findings suggest that adherence to the Mediterranean diet, as both a nutritional model and a lifestyle, may facilitate mindfulness-based approaches, including responsiveness to body signals, eating for physical rather than emotional reasons, and preventing excessive food consumption in response to external stimuli. Our study contributes to the literature by illuminating the influence of Mediterranean diet adherence on mindfulness-based eating behaviors among young adults, while also identifying the divergent roles of BMI in predicting these constructs.

This study has several limitations. The cross-sectional design precludes causal inference regarding the directionality of associations. The use of convenience sampling through online recruitment may have introduced selection bias, limiting generalizability to young adults with internet access and digital literacy. All data were self-reported, potentially subject to reporting and social desirability biases. Despite controlling for numerous covariates, residual confounding from unmeasured factors cannot be excluded.

## 5. Conclusions

Higher Mediterranean diet adherence was associated with higher intuitive eating and mindful eating among Turkish young adults. The divergent BMI associations—negative for intuitive eating, positive for mindful eating—suggest that these constructs capture distinct dimensions of eating behavior: physiological attunement versus cognitive control. For clinical practice, nutrition counselors might consider integrating Mediterranean diet promotion with intuitive eating principles, particularly for higher-BMI individuals who may benefit from restoring connection to internal hunger and satiety cues. Future research should employ longitudinal designs to establish temporal relationships, conduct randomized controlled trials to test whether Mediterranean diet interventions improve eating behavior outcomes, and explore potential mediators such as body image, weight stigma, and emotional regulation that may explain the observed associations.

## Figures and Tables

**Table 1:** Results of regression assumption tests for IES-2 and MEQ-30 models.

Assumption Test	IES-2 Result	MEQ-30 Result	Decision
Normality (Skewness)	0.374	−0.006	Acceptable (|value| < 1)
Normality (Kurtosis excess)	−0.379	1.34	IES-2: OK; MEQ-30: Marginal
Residual Normality (AD test)	AD = 7.61, *p* < 0.001	AD = 5.80, *p* < 0.001	Formal rejection (expected for *n* > 1000)
Homoscedasticity (BP test)	BP = 49.64, *p* < 0.001	BP = 30.21, *p* = 0.011	Both: Robust SE
Multicollinearity (VIF)	Max = 1.77	Max = 1.77	Acceptable (VIF < 5)
Independence (DW test)	DW = 1.80	DW = 1.90	Acceptable (1.5–2.5)
Linearity (Rainbow test)	F = 1.01, *p* = 0.418	F = 1.13, *p* = 0.032	IES-2: OK; MEQ-30: Marginal
Quadratic term test	F = 0.04, *p* = 0.846	F = 2.46, *p* = 0.117	Linear model sufficient

AD: Anderson–Darling; BP: Breusch–Pagan; DW: Durbin–Watson; VIF: Variance Inflation Factor. Robust SE: heteroscedasticity-consistent standard errors (HC3) used.

**Table 2:** Descriptive characteristics of participants (*N* = 2293).

Characteristic	*n*	% or Mean ± SD
**Demographic characteristics**		
Age (years), mean ± SD	2293	23.51 ± 3.27
**Gender**		
Female	1353	59.0
Male	940	41.0
**Marital status**		
Single	1911	83.3
Married	382	16.7
**Education level**		
University or higher	1894	82.6
High school or lower	399	17.4
**Employment status**		
Student	1132	49.4
Employed	821	35.8
Unemployed	338	14.8
**Socioeconomic status**		
**Perceived income level**		
Income < expenditure	428	23.8
Income = expenditure	933	52.0
Income > expenditure	434	24.2
**Health status**		
Chronic disease (yes)	234	10.2
BMI (kg/m^2^), mean ± SD	2293	22.93 ± 3.79
**BMI categories**		
Underweight (<18.5)	236	10.3
Normal weight (18.5–24.9)	1476	64.4
Overweight (25.0–29.9)	478	20.8
Obese (≥30.0)	103	4.5
**Lifestyle behaviors**		
Current smoker (yes) ^a^	807	35.2
Alcohol consumption (yes) ^b^	805	35.1
Regular physical activity (yes) ^c^	718	31.3
**Nutrition habits**		
**Number of daily meals**		
≤2 meals	855	37.3
3 meals	989	43.1
≥4 meals	437	19.6
Regular breakfast consumption (yes)	1347	58.7
**Daily water intake**		
≤9 glasses	1662	72.6
≥10 glasses	627	27.4

BMI: body mass index; SD: standard deviation. ^a^ Defined as smoking at least one cigarette per day. ^b^ Defined as any self-reported alcohol consumption, regardless of frequency or quantity. ^c^ Defined as engaging in physical activity on a regular basis. Turkish young adults aged 18–34 years.

**Table 3:** Mean scores of the Mediterranean Diet Adherence, Intuitive Eating Scale, and Mindful Eating Questionnaire of the research group.

Scale	*n* (%)	Mean ± SD	Min–Max	Range
**Mediterranean Diet Adherence Scale (MEDAS)**		5.83 ± 1.87	1.00–13.00	0–14.00
**Adherence to the Mediterranean Diet**				
Low (≤6 points)	1480 (64.5)			
Middle (7–8 points)	620 (27.0)			
High (≥9 points)	193 (8.4)			
**Intuitive Eating Scale 2 (IES 2)**		3.25 ± 0.59	1.76–5.00	1.00–5.00
Unconditional permission to eat		3.23 ± 0.61	1.00–5.00	1.00–5.00
Eating for physical rather than emotional reasons		3.29 ± 0.75	1.00–5.00	1.00–5.00
Eating based on hunger and satiety signals		3.24 ± 1.09	1.00–5.00	1.00–5.00
Body food choice compatibility		3.18 ± 1.11	1.00–5.00	1.00–5.00
**Mindful Eating Questionnaire (MEQ 30)**		82.64 ± 17.49	30.00–150.00	30.00–150.00
Disinhibition		14.25 ± 3.27	5.00–25.00	5.00–25.00
Emotional Eating		13.98 ± 3.58	5.00–25.00	5.00–25.00
Eating Control		10.95 ± 3.11	4.00–20.00	4.00–20.00
Focusing		13.27 ± 3.51	5.00–25.00	5.00–25.00
Eating Discipline		10.89 ± 2.74	4.00–20.00	4.00–20.00
Awareness		14.13 ± 3.56	5.00–25.00	5.00–25.00
Interference		5.22 ± 1.98	2.00–10.00	2.00–10.00

**Table 4:** Mean scores of the Intuitive Eating Scale and Mindful Eating Questionnaire according to adherence to the Mediterranean diet.

Scale Total and Sub-Dimensions		Adherence to the Mediterranean Diet					
		Low ^a^	Moderate ^b^	High ^c^	F	*p*	Post Hoc
**Intuitive Eating Scale-2 (IES-2)**		3.22 ± 0.59	3.28 ± 0.60	3.36 ± 0.60	6.59	<0.001	a < c
	Unconditional permission to eat	3.29 ± 0.60	3.15 ± 0.60	2.99 ± 0.62	28.13	<0.001	a > b, a > c, b > c
	Eating for physical rather than emotional reasons	3.26 ± 0.73	3.31 ± 0.77	3.37 ± 0.76	2.17	0.115	-
	Eating based on hunger and satiety signals	3.16 ± 1.08	3.35 ± 1.08	3.58 ± 1.11	17.26	<0.001	a < b, a < c, b < c
	Body–food choice compatibility	3.07 ± 1.10	3.32 ± 1.09	3.65 ± 1.15	30.46	<0.001	a < b, a < c, b < c
**Mindful Eating Questionnaire (MEQ-30)**		81.49 ± 17.78	84.36 ± 16.79	85.95 ± 16.68	9.73	<0.001	a < b, a < c
	Disinhibition	13.91 ± 3.26	14.71 ± 3.09	15.35 ± 3.47	25.53	<0.001	a < b, a < c, b < c
	Emotional Eating	13.51 ± 3.50	14.61 ± 3.45	15.51 ± 3.85	41.65	<0.001	a < b, a < c, b < c
	Eating Control	10.97 ± 3.13	10.94 ± 3.02	10.78 ± 3.18	0.33	0.717	-
	Focusing	13.14 ± 3.49	13.48 ± 3.52	13.61 ± 3.66	3.01	0.049	a < b
	Eating Discipline	10.77 ± 2.76	11.03 ± 2.70	11.36 ± 2.68	5.14	0.006	a < c
	Awareness	13.99 ± 3.62	14.35 ± 3.50	14.41 ± 3.31	2.98	0.051	-
	Interference	5.25 ± 1.96	5.24 ± 1.99	4.92 ± 2.04	2.39	0.092	-

Values are presented as Mean ± SD. ^a^: low adherence: MEDAS score ≤ 6 (*n* = 1480); ^b^: moderate adherence: MEDAS score 7–8 (*n* = 620); ^c^: high adherence: MEDAS score ≥ 9 (*n* = 193). One-way ANOVA with Tukey HSD post hoc test was used for group comparisons. The difference column shows significant pairwise comparisons (*p* < 0.05).

**Table 5:** Hierarchical multiple regression analysis predicting intuitive eating (IES-2 total score).

Variable		B	SE (Robust)	t	*p*	95% CI	Share of R^2^ (%)	R^2^	Adj. R^2^	ΔR^2^	F Change	AIC	ΔAIC
**(Intercept)**		3.611	0.186	19.38	<0.001 ***	[3.246, 3.977]	–						
**Block 1: Demographic**								0.006	0.005	–	–	3210.05	–
	Age	0.004	0.006	0.56	0.578	[−0.009, 0.016]	0.66						
	Gender (male)	0.001	0.034	0.04	0.966	[−0.065, 0.067]	4.36						
**Block 2: + Socioeconomic**								0.016	0.012	0.010 **	3.48 **	3202.67	−7.38
	Education (no university)	−0.087	0.040	−2.15	0.032 *	[−0.166, −0.007]	8.78						
	Employment (student)	0.006	0.040	0.14	0.889	[−0.073, 0.084]	0.68						
	Employment (not employed)	−0.034	0.048	−0.72	0.473	[−0.128, 0.059]	0.56						
	Income (low)	−0.020	0.035	−0.58	0.565	[−0.088, 0.048]	0.58						
	Marital status (married)	−0.039	0.051	−0.78	0.438	[−0.139, 0.060]	2.93						
**Block 3: + Health**								0.038	0.033	0.022 ***	19.66 ***	3167.57	−35.10
	Chronic disease (yes)	0.078	0.049	1.60	0.110	[−0.018, 0.174]	1.94						
	BMI (kg/m^2^)	−0.025	0.004	−5.68	<0.001 ***	[−0.033, −0.016]	40.85						
**Block 4: + Lifestyle**								0.043	0.036	0.005 *	2.91 *	3164.80	−2.77
	Smoking (yes)	−0.067	0.032	−2.08	0.038 *	[−0.130, −0.004]	4.45						
	Alcohol use (yes)	0.068	0.032	2.11	0.035 *	[0.005, 0.131]	4.00						
	Physical activity (no)	0.009	0.033	0.28	0.780	[−0.055, 0.073]	0.58						
**Block 5: + Nutrition habits**								0.053	0.045	0.010 ***	6.20 ***	3152.12	−12.68
	Daily meals (*n*)	−0.034	0.014	−2.52	0.012 *	[−0.061, −0.008]	3.87						
	Breakfast (yes)	0.109	0.031	3.58	<0.001 ***	[0.049, 0.169]	14.31						
	Water intake (glasses/day)	0.003	0.004	0.78	0.434	[−0.005, 0.011]	0.74						
**Block 6: + MEDAS**								0.058	0.049	0.005 **	8.49 **	3145.57	−6.55
	MEDAS score	0.023	0.008	2.92	0.004 **	[0.008, 0.039]	9.72						

*n* = 1750. Hierarchical multiple regression with robust standard errors (HC3) due to heteroscedasticity. Final model: R^2^ = 0.058, adjusted R^2^ = 0.049, F_(16, 1733)_ = 6.61, *p* < 0.001. Reference categories: Female, University education, Employed, Income adequate/high, Single, No chronic disease, Non-smoker, No alcohol, Physically active, No breakfast. CI: confidence interval. Share of R^2^ calculated using the Lindeman–Merenda–Gold (LMG) method for relative importance. The Wald test was used for MEDAS contribution (robust): F_(1, 1733)_ = 8.53, *p* = 0.004. * *p* < 0.05, ** *p* < 0.01, *** *p* < 0.001.

**Table 6:** Hierarchical multiple regression analysis predicting mindful eating (MEQ-30 total score).

Variable		B	SE (Robust)	t	*p*	95% CI	Share of R^2^ (%)	R^2^	Adj. R^2^	ΔR^2^	F Change	AIC	ΔAIC
**(Intercept)**		57.571	5.750	10.1	<0.001 ***	[46.302, 68.841]	–						
**Block 1: Demographic**								0.006	0.004	–	–	14,994.79	–
	Age	−0.071	0.199	−0.36	0.721	[−0.460, 0.319]	1.11						
	Gender (male)	−4.376	1.005	−4.36	<0.001 ***	[−6.345, −2.407]	14.15						
**Block 2: + Socioeconomic**								0.017	0.013	0.011 **	4.04 **	14,984.63	−10.16
	Education (no university)	−1.717	1.251	−1.37	0.170	[−4.170, 0.735]	2.69						
	Employment (student)	3.200	1.188	2.69	0.007 **	[0.872, 5.527]	9.73						
	Employment (not employed)	−1.221	1.343	−0.91	0.363	[−3.853, 1.410]	1.56						
	Income (low)	1.745	1.052	1.66	0.097	[−0.316, 3.807]	2.97						
	Marital status (married)	0.961	1.483	0.65	0.517	[−1.946, 3.869]	1.2						
**Block 3: + Health**								0.044	0.039	0.028 ***	25.05 ***	14,938.95	−45.68
	Chronic disease (yes)	3.315	1.352	2.45	0.014 *	[0.665, 5.966]	7.50						
	BMI (kg/m^2^)	0.768	0.126	6.10	<0.001 ***	[0.521, 1.015]	29.66						
**Block 4: + Lifestyle**								0.050	0.043	0.006 *	3.42 *	14,934.64	−4.31
	Smoking (yes)	−1.021	0.921	−1.11	0.267	[−2.826, 0.783]	1.38						
	Alcohol use (yes)	2.475	0.904	2.74	0.006 **	[0.703, 4.246]	5.22						
	Physical activity (no)	−0.754	0.983	−0.77	0.443	[−2.680, 1.172]	0.71						
**Block 5: + Nutrition habits**								0.056	0.048	0.006 **	3.90 **	14,928.87	−5.77
	Daily meals (*n*)	0.900	0.398	2.26	0.024 *	[0.120, 1.680]	4.77						
	Breakfast (yes)	0.446	0.889	0.50	0.616	[−1.297, 2.189]	1.5						
	Water intake (glasses/day)	0.076	0.117	0.65	0.515	[−0.153, 0.306]	1.87						
**Block 6: + MEDAS**								0.062	0.054	0.006 **	11.13 **	14,919.67	−9.20
	MEDAS score	0.776	0.241	3.22	0.001 **	[0.304, 1.247]	13.61						

*n* = 1750. Hierarchical multiple regression with robust standard errors (HC3) due to heteroscedasticity (Breusch–Pagan test: BP = 30.21, *p* = 0.011). Final model: R^2^ = 0.062, adjusted R^2^ = 0.054, F_(16, 1733)_ = 7.21, *p* < 0.001. Reference categories: Female, University education, Employed, Income adequate/high, Single, No chronic disease, Non-smoker, No alcohol, Physically active, No breakfast. CI: confidence interval. Share of R^2^ calculated using the Lindeman–Merenda–Gold (LMG) method for relative importance. The Wald test was used for MEDAS contribution (robust): F_(1, 1733)_ = 10.38, *p* = 0.001. * *p* < 0.05, ** *p* < 0.01, *** *p* < 0.001.

## Data Availability

The data presented in this study are available on request from the corresponding author due to ethical and privacy restrictions.
